# Defluorination of monofluorinated alkane by *Rhodococcus* sp. NJF-7 isolated from soil

**DOI:** 10.1186/s13568-024-01729-w

**Published:** 2024-06-06

**Authors:** Meng Yan, Zhaozhao Gao, Xingjia Xiang, Qing Wang, Xin Song, Yucheng Wu, Frank E. Löffler, Jun Zeng, Xiangui Lin

**Affiliations:** 1 Anhui Province Key Laboratory of Wetland Ecosystem Protection and Restoration, School of Resources and Environmental Engineering, Anhui University, Hefei, 230601 China; 2 State Key Laboratory of Soil and Sustainable Agriculture, Institute of Soil Science, Chinese Academy of Sciences, Beijing East Road, 71, Nanjing, 210008 China; 3 Department of Civil and Environmental Engineering, Department of Microbiology, Department of Biosystems Engineering and Soil Science, University of Tennessee, Knoxville, TN 37996 USA; 4 Department of Biology and Biochemistry, Institute of Soil Science, Chinese Academy of Sciences, Beijing East Road, 71, Nanjing, 210008 People’s Republic of China

**Keywords:** Microbial defluorination, *Rhodococcus*, Fluorinated alkane, pH, Fluoride toxicity

## Abstract

Microbial degradation of fluorinated compounds raised significant attention because of their widespread distribution and potential environmental impacts. Here, we report a bacterial isolate, *Rhodococcus* sp. NJF-7 capable of defluorinating monofluorinated medium-chain length alkanes. This isolate consumed 2.29 ± 0.13 mmol L^− 1^ of 1-fluorodecane (FD) during a 52 h incubation period, resulting in a significant release of inorganic fluoride amounting to 2.16 ± 0.03 mmol L^− 1^. The defluorination process was strongly affected by the initial FD concentration and pH conditions, with lower pH increasing fluoride toxicity to bacterial cells and inhibiting enzymatic defluorination activity. Stoichiometric conversion of FD to fluoride was observed at neutral pH with resting cells, while defluorination was significantly lower at reduced pH (6.5). The discovery of the metabolites decanoic acid and methyl decanoate suggests that the initial attack by monooxygenases may be responsible for the biological defluorination of FD. The findings here provide new insights into microbial defluorination processes, specifically aiding in understanding the environmental fate of organic semi-fluorinated alkane chemicals.

## Introduction

The discovery of beneficial properties in organic fluorochemicals has led to increased incorporation of elemental fluorine into the production of commercial goods, such as pesticides, materials, and pharmaceuticals, which are widely employed and subsequently released into the environment (Pétré et al. 2021; Wang et al. 2017). Synthetic fluorinated compounds have raised significant environmental concerns because of their persistence and potential risks to human and ecosystem health (Lau et al. 2007; Wang et al. 2022; Xiao 2017; Yin et al. 2022). These compounds’ recalcitrance is primarily attributable to the strength of carbon–fluorine (C–F) bonds (Natarajan et al. 2005; O’Hagan 2008), and it is believed that a fundamental understanding of microbial defluorination processes is crucial for comprehending their fate in the environment. Microbial defluorination faces significant challenges (Che et al. 2021; Alexandrino et al. 2018). There are only ~ 20 monofluorinated compounds in nature, such as fluoroacetate, nucleocidin, and some fluorinated fatty acids (Carvalho and Oliveira 2017; Walker and Chang 2014), and microbes may have had insufficient time to evolve specific enzymes for degrading complex fluorinated xenobiotics (Wackett 2021, 2022a). Further, the released fluoride can be toxic, requiring microbes to possess a specialized system for fluoride export (Baker et al. 2012; Johnston and Strobel 2020; Li et al. 2013; Zhu et al. 2019). Nevertheless, the existence of naturally occurring simple organofluorides produced by certain plants or actinomycetes-like microorganisms suggests that at least some microorganisms have evolved biological systems to catabolize these compounds and cleave C–F bonds (Carvalho and Oliveira 2017).

Various studies have demonstrated a negative relationship between defluorination difficulty and the number of fluorine substitutions (Che et al. 2021; Khan et al. 2023). The degradation of fluorinated compounds possessing low degrees of fluorination was initially investigated using fluoroacetate, a common naturally fluorinated fatty acid (Kelly 1965). Diverse bacteria belonging to the genera *Pseudomonas*, *Delftia*, *Burkholderia*, *Comamonas*, and *Variovorax* can degrade fluoroacetate (Donnelly and Murphy 2009; Kurihara et al. 2003; Leong et al. 2016). For example, *Comamonas testosteroni* MFA1 and *Variovorax paradoxus* MFA10 can utilize fluoroacetate as their sole carbon source while simultaneously releasing fluoride (Alexandrino et al. 2018); however, they showed no degradation activity on di- or tri-fluoroacetate. Similarly, fluorinated aromatics with low degrees of fluorination, such as 4-fluorobenzoate, 4-fluorotoluene, and 2-fluorobenzoate, can be utilized as carbon and energy sources (Kiel and Engesser 2015; Seong et al. 2019). In contrast, the defluorination of highly fluorinated organics can also be achieved, but this metabolic pathway appears to be restricted to highly specialized microbial communities (Che et al. 2021; Jin et al. 2023; Yu et al. 2022).

The microbial degradation of alkanes has been widely studied (Liu et al. 2022; Wang et al. 2019), while reports on degrading fluorinated alkanes via microbe remain scarce. Previous studies suggested that bacteria capable of degrading chlorinated or brominated alkanes may be unable to degrade fluorinated alkanes because of the strength of C–F bonds (Cui et al. 2018; Nichols et al. 2013). However, recent studies indicated that *Pseudomonas* sp. strain 273, originally isolated as a dechlorinating bacterium, exhibited defluorination activity on the medium-chain fluoroalkane, 1-fluorodecane (FD) (Wischnak et al. 1998; Xie et al. 2020, 2022, 2023), indicating this bacterium possesses an enzyme system that can attack terminally fluorinated alkanes. The diversity and environmental distribution of bacteria capable of attacking fluorinated alkane remains unclear, and whether defluorinating bacteria could be obtained directly using fluorinated alkane is uncertain. The present study sought to isolate bacteria from soil samples with fluoroalkane as a carbon source. The present work unveiled the substantial impact of pH on microbial defluorination, affecting fluoride toxicity in cells and altering the stoichiometric conversion of fluorinated compounds to fluoride through enzymatic processes. The findings contribute to a fundamental understanding of bacterial defluorination processes and have implications for the fate of fluorinated organic compounds.

## Materials and methods

### Chemicals and media

FD (purity, > 97%) was purchased from SynQuest Lab, Inc. (Alachua, FL, USA). Reagent grade or higher purity solvents, such as *n*-hexane and acetone, were obtained from Tedia (Fairfield, OH, USA). The completely synthetic minimal medium (MM) used for the degradation experiments comprised the following components per liter: MgSO_4_·2H_2_O 0.2 g; CaCl_2_·2H_2_O 20 mg; FeSO_4_·7H_2_O 10 mg; KH_2_PO_4_ 0.4 g; Na_2_HPO_4_ 0.6 g; MnSO_4_ 20 mg; NaNO_3_ 1 g; NH_4_Cl 0.6 g. The three compounds containing only milligram quantities were prepared as a 100-fold concentrate before being mixed with other substances for accuracy. Phosphates were prepared as a 10-fold concentrate, sterilized separately, and mixed with the culture medium to prevent precipitation. Luria–Bertani (LB) medium was composed of 1% peptone, 0.5% yeast extract, and 1% NaCl, adjusting the pH with HCl or NaOH.

### Isolation of a novel FD-degrading bacterium

The bacterium was isolated from soil collected at a depth of > 6 m from a chemical plant in Suzhou, Jiangsu Province, China (31°17′ N, 120°24′ E). The soil was manually homogenized, and 20 g was added to a flask containing 50 mL of MM. The flask received 5 mmol L^− 1^ FD, and incubation was performed at 28 °C for 4 weeks on a rotary shaker (160 rpm). At the end of enrichment, an aliquot (100 µL) of diluted sample was spread on a double-layer plate and incubated at a constant temperature (25 °C) for 2 weeks. The bilayer plates were prepared using a previously described method with modifications Zeng et al. (2010). Briefly, the overlayer was mixed with 0.25 mL of an acetone solution of FD (40 µL mL^− 1^) with 5 mL MM medium containing 1% agar. After the contents were mixed, the overlayer was immediately poured on an underlayer of MM solidified 1.5% agar. Yeast extract (0.05%, v/v) was provided in an overlayer to stimulate bacterial growth. Colonies were selected from the plate and purified by repeated streaking on a new bilayer plate. Pure cultures were routinely cultured in the presence of 5 mmol L^− 1^ of FD to maintain their degrading capability.

### Identification of the FD-degrading isolate

The isolate, designated strain NJF-7, proliferated in a LB medium and was used for genomic DNA extraction with MolPure Bacterial DNA Kit (Yeasen Biotechnology Co., Ltd, Shanghai, China). The 16 S rRNA gene was amplified using primer 27 F and 1492R (Weisburg et al. 1991). The PCR amplification conditions were: initial cycle at 95 °C for 5 min, followed by 35 cycles including denaturation at 95 °C for 30 s, annealing at 58 °C for 30 s, extension at 72 °C 90 s, and a final cycle at 72 °C for 7 min. The PCR products were purified and sent for sequencing. The 16 S rRNA gene sequence of NJF-7 was aligned with the sequences in the NCBI database (https://blast.ncbi.nlm.nih.gov/Blast.cgi), and a phylogenetic tree was constructed using MEGA (Version 11). The selected physiological and biochemical properties of the isolate were determined using classical microbiological experimental methods (McDevitt 2009; Smith and Hussey 2005). Strain NJF-7 was deposited in the China General Microbiological Culture Collection with CGMCC 1.19448 as a reference.

The 16 S rRNA gene sequence of NJF-7 was deposited in GenBank (accession number OR125658).

#### Defluorination of FD by strain NJF-7

The degradation was performed with 5 mmol L^− 1^ of FD as the sole carbon source using 10% bacterial inoculation (v/v) in a 5 mL MM medium incubated at 28 °C at 160 rpm. NJF-7 cells grown in LB medium (*OD*_600_ of 0.6 ~ 0.8) were harvested by centrifugation (4000 rpm, 5 min) and washed three times with MM, and resuspended in an equal volume for bacterial inoculation. The effects of the applied concentration of FD were tested at 0.5, 2.5, and 5 mmol L^− 1^, while pH effects were tested at 5.5, 6.5, 7.2, and 8.5, respectively. The treatments were conducted in triplicates.

The entire vessel was sacrificed, and the remaining FD was extracted three times with *n*-hexane and quantified using gas chromatography–mass spectrometry (GC–MS). Fluoride release was determined using ion chromatography (IC). For the preparation of IC samples, 1 mL of culture was collected and filtered using a 0.22 μm hydrophilic filter membrane. The release of fluoride was qualified and quantified using the peak times and peak areas from the ion chromatograms. The defluorination ratio is calculated using the formula below.

$$ \text{Defluorination rati}\text{o}= \frac{\text{Floride released from FD (mM)}}{\text{initial added FD (mM)}} \times 100\%$$

#### Growth yields of strain NJF-7 with decane and FD

NJF-7 was grown in 60 mL glass vials containing 10 mL of medium with decane and FD (5 mM), respectively. Six replicates were conducted for a total of twelve vials. After 6 days of shock incubation, half of the samples were subjected to whole-sample extraction to quantify the residual substrate. The remaining half of the samples were used to determine cell production via the Bradford protein assay, as described in Xie et al. (2020).

### Effect of fluoride on bacterial growth and survival at different pH values

The effect of fluoride on cell growth and survival was investigated at pH 7.2 and pH 6.5, as pH can influence fluoride toxicity (Johnston and Strobel 2020). Bacterial growth in 100 mL MM medium containing 0.3% (w/v) glucose was determined periodically using *OD*_600_, with varying concentrations of NaF (1, 5, and 10 mmol L^− 1^). For survival assessments, bacterial cells (10% inoculation, v/v) were exposed to NaF (5, 10, and 200 mmol L^− 1^) for 24 h, and colony-forming units were determined using serial dilution and plating on LB medium. The controls were conducted without adding NaF, and all tests were performed in triplicate.

#### Defluorination of FD with resting cells and identification of potential metabolites

Cells of NJF-7, cultivated in 1 L decane-containing MM medium for 3 days, were harvested by centrifugation, washed three times, and, respectively, resuspended in 100 mL of MM medium at pH 6.5 and pH 7.2. These suspensions were used for resting cell experiments, where 10 mL of concentrated cells were incubated with 5 mmol L^− 1^ FD at 28 °C for 20 h. Control experiments were set as resting cells incubated for the same amount of time in the absence of FD.

The metabolites from the above two treatments were analyzed using an identical method. A volume of 10 mL of incubated solution was collected and acidified to pH < 2 using sulfuric acid. The mixture was extracted twice using 10 mL of ethyl acetate. The organic phase was collected and placed into a 100 mL round-bottomed flask and rotary evaporated to remove the solvent. A volume of 12 mL of sulfuric acid-methanol (1:10, v/v) solution was added, tightly capped, vortexed, shocked, and heated at 70 °C for 1 h. After cooling in an ice bath, 12 mL of a 1:1 mixture of hexane and methyl tert-butyl ether was added to extract the methyl-esterified metabolite via shaking. The upper hexane phase was aspirated into another clean test tube and concentrated to dryness using nitrogen blowing in an electrically heated dry bath at 40 °C. The methyl fatty acid ester was then dissolved in 500 µL of *n*-hexane, transferred to the injection bottle, and analyzed using GC–MS.

### Analytical procedures

GC–MS was performed using a TQ8050 MDGCMSMS instrument (Shimadzu, Kyoto, Japan) with a UA-5MS column (30 m length, 0.25 mm inner diameter, 0.25 μm film thickness) and helium as the carrier gas at a flow rate of 3.0 mL min^− 1^. The sample, injected at a ratio of 20:1, was subjected to an oven at 70 °C for 2 min, followed by a ramp from 70 to 310 °C at a rate of 10 °C min^− 1^ over a total program time of 32 min. The ion source temperature was maintained at 230 °C, and full scans were obtained between 50 and 550 *m/z*. IC was performed on an ICS-5000 + dual system (Thermo Fisher Scientific, Waltham, MA, USA) using an anionic AS19-equivalent column, AERS_4mm suppressor, and 35.0 mmol L^− 1^ hydroxide eluent at a flow rate of 1 mL min^− 1^. The statistical analysis for differences in FD and fluoride release among treatments was performed using the Student’s *t*-test and one-way analysis of variance (ANOVA) with SPSS Statistics software (Version 19).

## Results

### Isolation of FD-degrading bacterium

**Fig. 1:**
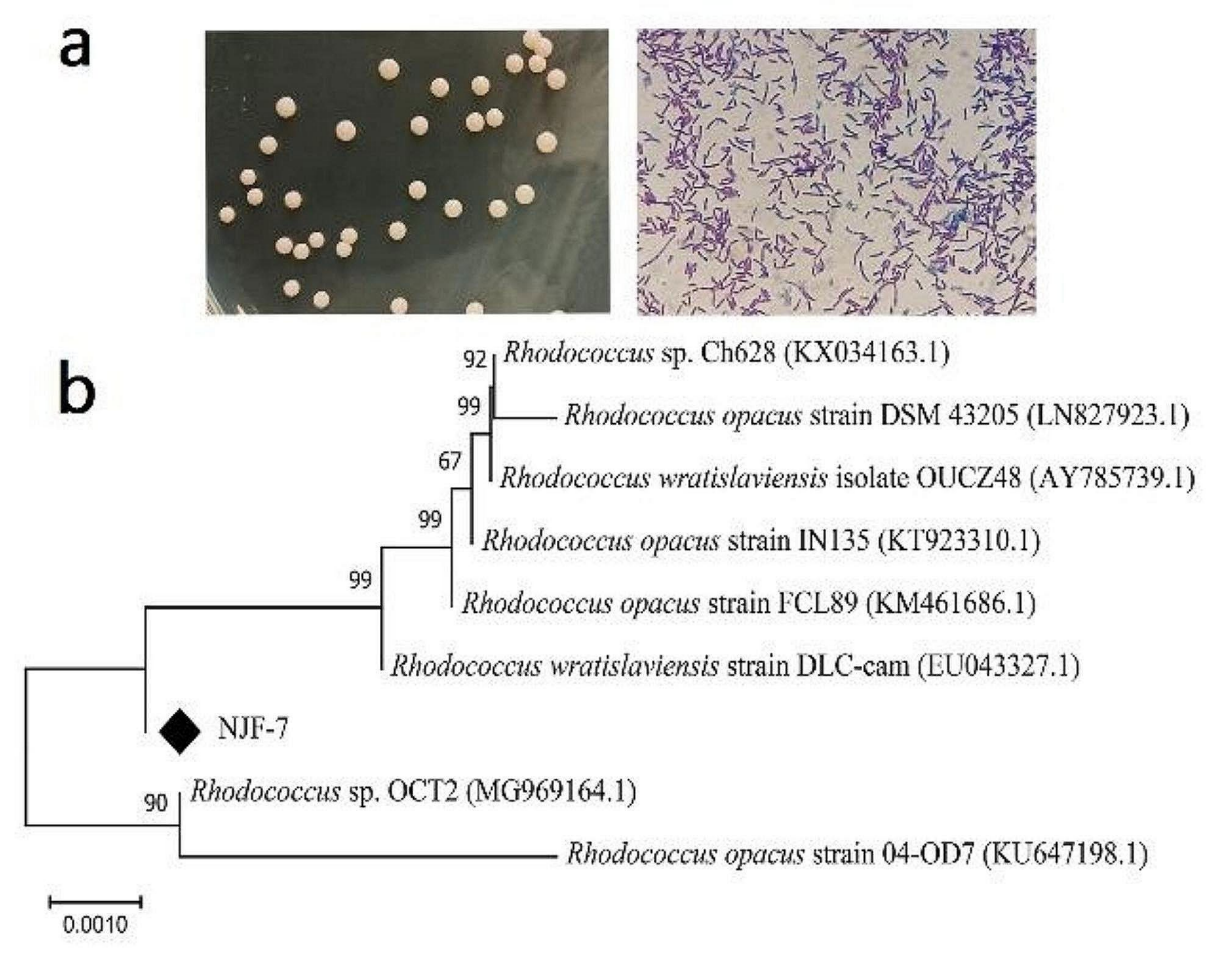
Monofluorinated alkane-defluorinating bacterium *Rhodococcus* sp. NJF-7 isolated from soils. **a** Photographs of bacterial colony and cell morphology. **b** Neighbor-joining phylogenetic tree of the isolate based on 16 S rRNA gene sequences; the numbers at the branch points indicate bootstrap values (%) for 1,000 replicates

The FD-degrading bacterium NJF-7 was isolated utilizing FD as the sole carbon source. It was Gram-positive and had pale orange–red, round, smooth, and moist appearance colony and short-rod-shaped cell morphologies (Fig. 1a). The isolate showed strong catalase activity but was unable to utilize sodium citrate or hydrolyze starch and cellulose (Table 1). Phylogenetic analysis indicated that the 16 S rRNA gene sequence of strain NJF-7 had 100% identity to that of *Rhodococcus wratislaviensis* strain DLC-cam (Fig. 1b).

**Table 1:** Selected physiological and biochemical characterization of *Rhodococcus* sp. NJF-7

Characteristics	Results
Gram stain	+
Spore stain	−
Methyl red test	−
Voges–Proskauer reaction	−
Sodium citrate	−
Starch hydrolysis	−
Cellulose hydrolysis	−
Catalase reaction	+

“+” is positive “−” is negative

### Defluorination of FD by strain NJF-7

**Fig. 2:**
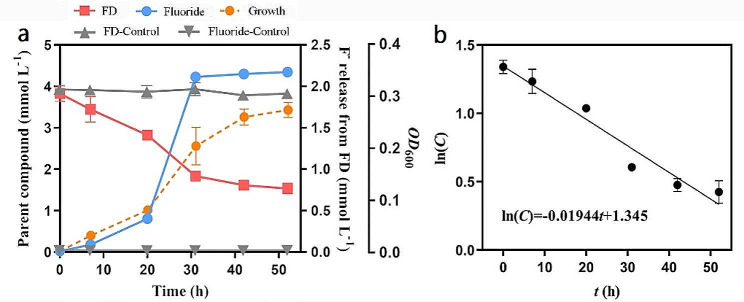
Defluorination of 1-fluorodecane by *Rhodococcus* sp. NJF-7. **a** Consumption of substrate and release of fluoride during a 52 h incubation period. Growth is indicated by the increased optical density (*OD*_600_). The control experiment contained only 5mmol L^− 1^ FD. **b** Defluorination ratio within each incubation interval. The results represent the mean of three replicate cultures with error bars indicating the standard deviation

**Fig. 3:**
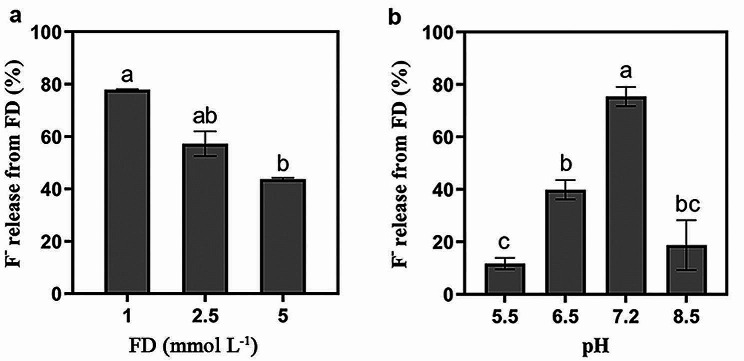
Effects of applied 1-fluorodecane concentration (**a**) and pH conditions (**b**) on defluorination by *Rhodococcus* sp. NJF-7. The mean and standard deviation of triplicate experiments are shown. Different letters indicate significant differences among treatments (*P* < 0.05, ANOVA)

The release of fluoride was recognized as a defluorination indicator (Bygd et al. 2022). The biodegradation of FD, with FD as the sole carbon source, using strain NJF-7 at sufficient oxygen (pH = 6.8) resulted in the consumption of substrate in the amount of 2.29 ± 0.13 mmol L^− 1^ after 52 h of incubation, accounting for 45.8 ± 2.6% of the initially applied concentration. Inorganic fluoride was gradually released during FD degradation, with 2.16 ± 0.03 mmol L^− 1^ (43.2 ± 0.6%) fluoride detected after the incubation period (Fig. 2a). Strain NJF-7 produced 1.23 ± 0.22 µg of protein per µmol carbon consumed using FD as the sole carbon source (Table 2). The degradation of FD by strain NJF-7 followed a first-order kinetic model, and the fitted curve is shown in Fig. 2b (C represents the residual concentration of FD). The specific rate constant is 0.01944, *R*^*2*^ = 0.94. The degradation half-life (*t*_1/2_) of FD was 35.7 h, and the maximum degradation rate during the degradation process was 0.99 mmol L^− 1^ h^− 1^. Three days after the incubation period, the defluorination amounts were 77.9 ± 0.1%, 57.3 ± 4.8%, and 43.7 ± 0.5% at 1, 2.5, and 5 mmol L^− 1^ of FD, respectively, at natural pH (*P* < 0.05, Fig. 3a). In addition, bacterial defluorination was significantly affected by pH, with a maximum fluoride release of 1.89 ± 0.09 mmol L^− 1^ at pH 7.2. In contrast, only 0.29 ± 0.05 mmol L^− 1^ was released at pH 5.5 (*P* < 0.05, Fig. 3b).

**Table 2:** *Rhodococcus* sp. NJF-7 growth yields with decane and 1-fluorodecane

Carbon substrate	Decane	1-fluorodecane
Substrate consumed (mmol/L)	1.70 ± 0.11	1.20 ± 0.13
Carbon consumed (µmol)	169.53 ± 10.87	119.68 ± 13.21
Protein (µg/µmol carbon)	4.11 ± 0.20	1.23 ± 0.22

### Effects of fluoride on the growth and survival of cells

**Fig. 4:**
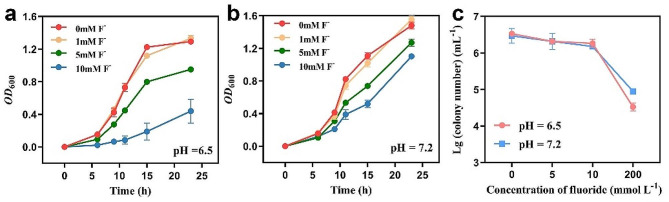
Effects of fluoride concentrations on the growth (**a** and **b**) and survival (**c**) of *Rhodococcus* sp. NJF-7 at pH 5.6 and pH 7.2 conditions. The number of colonies in panel (c) was logarithmically transformed. Error bars depict the standard deviation of triplicate measurements

A concentration of 1 mmol L^− 1^ fluoride did not significantly affect bacterial growth, but 10 mmol L^− 1^ fluoride remarkably inhibited growth, and the cell density (*OD*_600_) decreased by 66.0% and 22.6% at pH 6.5 and pH 7.2, respectively (Fig. 4a and b). Meanwhile, experiments on fluoride exposure showed that the number of NJF-7 colonies was 1.21 ~ 4.80 × 10^6^ per mL at 5 and 10 mmol L^− 1^ fluoride, but the number of colonies dropped to 2.50 ~ 9.50 × 10^3^ per mL at 200 mmol L^− 1^ fluoride, the toxicity of fluoride ions was enhanced by pH, and a stronger impact occurred at pH 6.5 (*P* < 0.05, Fig. 4c).

### Defluorination of FD by resting cells and potential metabolites

**Fig. 5:**
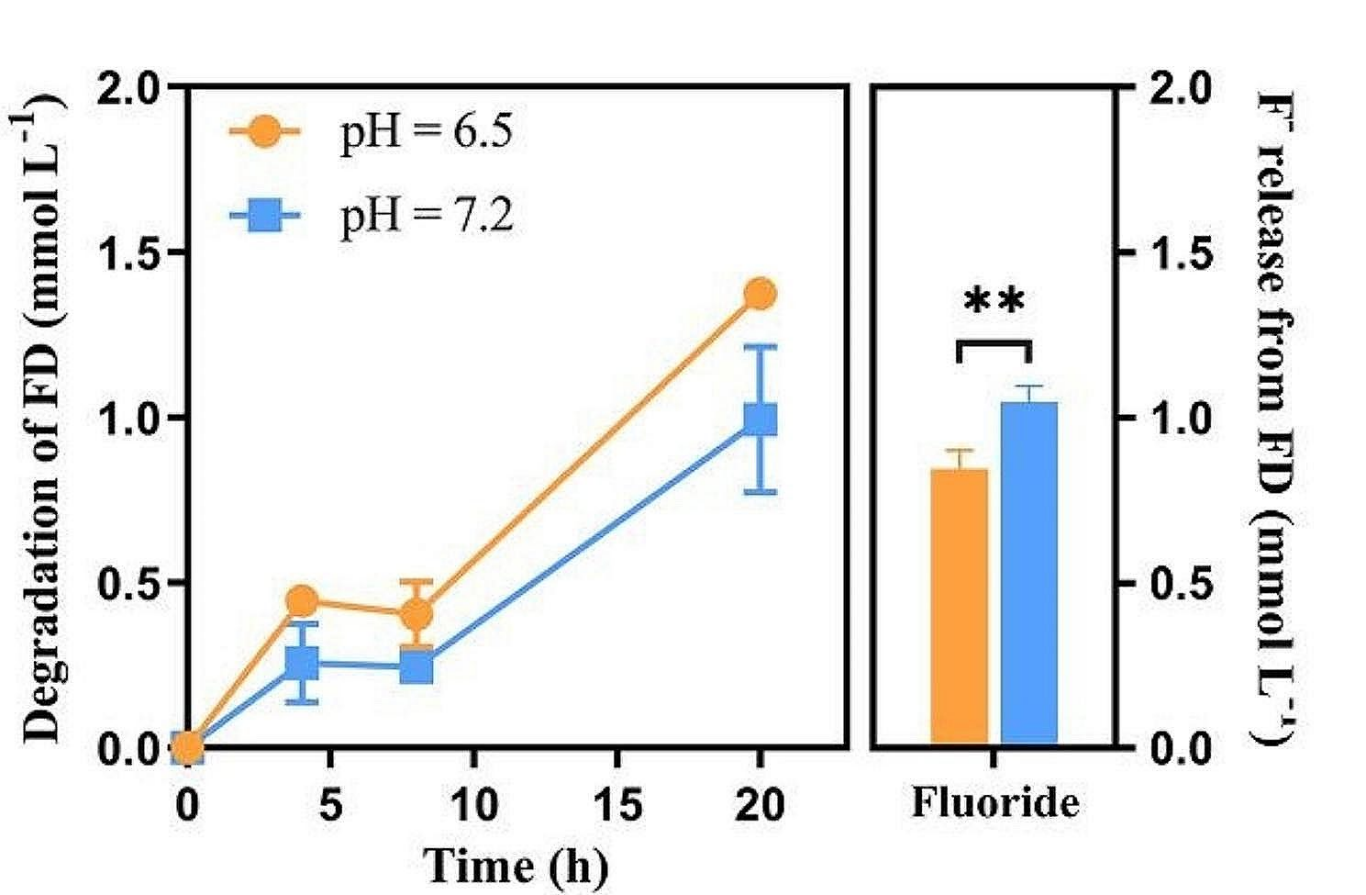
Defluorination of 1-fluorodecane with resting cells by *Rhodococcus* sp. NJF-7 at pH 6.5 and 7.2. The results are mean values measured in triplicate with error bars indicating standard deviation. Significant differences between treatments were determined using the Student’s *t*-test (**: *P* < 0.01)

Resting cells are used as catalysts for biotransformation; these cells are metabolically active but do not grow, minimizing growth-induced side effects (Diao et al. 2022; Rodríguez M et al. 2021). The NJF-7 resting cells consumed 1.37 ± 0.03 mmol L^− 1^ of FD after 20 h of incubation at pH 6.5, accounting for 27.5 ± 0.6% of the initially applied concentration. They released 0.85 ± 0.06 mmol L^− 1^ of fluoride, representing a conversion of 16.9 ± 1.1% of FD into fluoride. In contrast, 0.99 ± 0.22 mmol L^− 1^ of FD was consumed at pH 7.2, accounting for 19.8 ± 4.4% of the initially applied concentration; they released 1.05 ± 0.05 mmol L^− 1^ of fluoride (20.9 ± 0.98%), representing almost complete defluorination after 20 h of incubation. A significant difference in fluoride release was observed between the two pH conditions (*P* < 0.01) (Fig. 5).

Since alkanes are susceptible to attack by monooxygenases, it is assumed that the potential byproducts of FD degradation should be the corresponding alcohols, fluoroalcohols, carboxylic acids, fluorocarboxylic acids, etc. For the resting cell group, GC–MS analysis showed that methyl decanoate eluted at 10.38–10.58 min (m/z 186, M^+^) and the detected fragment ions included 155 (M^+^ − O − CH_3_), 143 (M^+^ − 2CH_2_ − CH_3_), 87 (M^+^ − 6CH_2_ − CH_3_), and 74 (M^+^ − 7CH_2_ − CH_3_ + 1) (Fig. 5, green line). These may be formed when monooxygenase attacks the fluorinated terminal carbon, leading to oxygenated dehydrogenation. Non–methyl derivatized decanoic acid (m/z 173, M^+^+H) was also eluted at 12.83–13.01 min; the fragment ions were similar to methyl decanoate. In contrast, the characteristic peaks of methyl decanoate and non–methyl derivatized decanoic acid did not appear in the control, suggesting that they are products of FD metabolism and not produced from other pathways (Fig. 5, red line).

## Discussion

Here, we demonstrate the potential of *Rhodococcus* sp. strain NJF-7 as a novel bacterium capable of degrading fluoroalkanes, with the degradation of FD leading to fluoride release (Fig. 2). Members of the *Rhodococcus* genus are widely distributed in soil, water, and sediment (Hassanshahian et al. 2013; Mikolasch et al. 2015), with an extensive metabolic versatility and unique degradation capabilities for various organic compounds, including polychlorinated biphenyls, polycyclic aromatic hydrocarbons, nitriles, and aliphatic hydrocarbons (Martinkova et al. 2009), which are likely associated with its large genome (up to 10 Mbp) and significant degree of functional redundancy (Zampolli et al. 2019). They are frequently isolated from petroleum-contaminated environments and have been reported as capable of utilizing various alkanes as carbon and energy sources (Cappelletti et al. 2015; Castro et al. 2016; de Carvalho et al. 2009). The production of biosurfactants and the presence of a hydrophobic cell surface are essential for the degradation of hydrophobic compounds by *Rhodococcus* strains (Ivshina et al. 2016). Bacteria in the *Rhodococcus* genus can degrade halogenated compounds such as decabromodiphenyl ether and chlorobenzoate, and the present study reports for the first time the biodegradation of fluorinated alkanes, extending the reported degradation capabilities of this genus (Emelyanova et al. 2023; Huang et al. 2017).

Bacterial defluorination was impacted significantly by the FD concentration and pH conditions (Fig. 3). The effect of substrate concentration aligned with a previous biodegradation study (Wang et al. 2010), indicating that elevated substrate concentrations resulted in overloading of functional enzymes. In addition, an abundance of insoluble substrates may stimulate the production of compounds such as biosurfactants, exacerbating bacterial metabolic burden (Rojo 2009). The pH affects not only bacterial growth but also closely influences fluoride toxicity, with higher toxicity observed at lower pH values (Fig. 4). Fluoride toxicity is linked closely to pH (Liao et al. 2017). In acidic environments, fluoride released by defluorination dynamically combines with H^+^ in water to form a weak acid, hydrofluoric acid. Hydrofluoric acid readily penetrates cell membranes and dissociates into H^+^ and F^−^ in the near-neutral cytoplasm, elevating the intracellular fluoride concentration (Ji et al. 2014; Marquis et al. 2003). Failure to promptly expel intracellular fluoride by microbial cells leads to its accumulation, which can then bind to the metal centers in crucial enzymes such as ATPases and pyrophosphatases, hindering intracellular ATP synthesis and impacting bacterial growth and metabolism (Liao et al. 2017; Wackett 2022b). Although fluoride can impact bacterial growth at intracellular micromolar levels, fluoride transport channels can maintain the intracellular fluoride concentration at low levels (Baker et al. 2012; Stockbridge et al. 2013). Ji et al. (2014) observed that bacterial cells exposed to 0.5 mmol L^− 1^ F^−^ for 16 h did not show reduced survival. However, this capacity is limited because fluoride induces bacterial death at the higher concentrations observed here (Fig. 4c), which aligns with previous studies (Calero et al. 2022; Li et al. 2013).

**Fig. 6:**
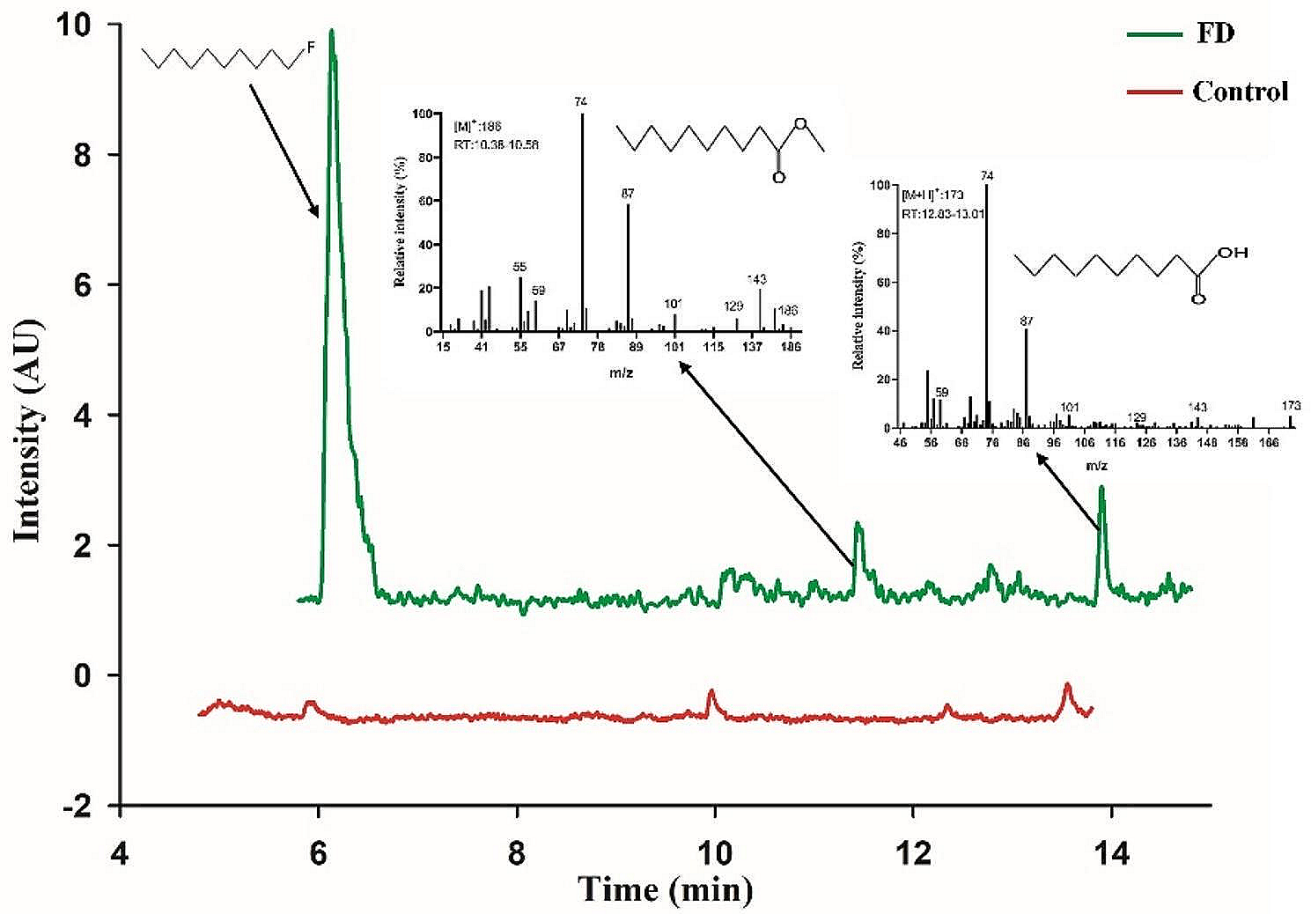
Mass spectrometry of methyl derivatives of FD metabolic intermediates in *Rhodococcus* sp. NJF-7 (pH 7.2). FD treatment indicates chromatograms of resting cells incubated with FD (Green). Control treatment indicates the resting cells incubated in the absence of FD (Red)

The results in the resting cell experiment indicated that, compared with neutral conditions, FD was readily degraded at lower pH but released less fluoride (Fig. 5). It was reported that specific enzymes can catalyze biological defluorination directly by breaking C–F bonds or indirectly via transforming fluorinated compounds to produce unstable intermediates that result in the spontaneous release of fluoride (Che et al. 2021; Kiel and Engesser 2015; Tiedt et al. 2016). GC–MS analysis showed that decanoic acid (CH_3_(CH_2_)_8_COOH) and methyl decanoate (CH_3_(CH_2_)_8_COOCH_3_) were detected in the resting cell treatment compared with control (Fig. 6). The results indicated that NJF-7 may attack the fluorine terminus through monooxygenase (Xie et al. 2020). FD was converted to the corresponding alcohols, which were further oxidized to the corresponding carboxylic acids. Fluoride was spontaneously eliminated during this process. However, the fluorination product produced by strain NJF-7 attacking the -CH_3_ terminus was not found.

In conclusion, the present study expands the substrate range of *Rhodococcus* spp. to fluorinated alkanes and identifies strain NJF-7 as a novel bacterium capable of degrading fluoroalkanes. The findings indicate that lower pH inhibits cell growth and enzyme activity as well as enhances fluoride toxicity. In addition, this study suggested that the biological defluorination of FD may be caused by monooxygenases attacking the fluorine-terminated carbon. These findings contribute to the understanding of microbial defluorination processes and the environmental fate of fluorinated chemicals. However, further research is needed to clarify the fundamental mechanisms of defluorination, especially functional genes and enzymes.

## Data Availability

All data generated and analyzed during this study are included in this published article.
